# Pancreatic Solid Pseudopapillary Tumor Associated with Elevated DHEA and Testosterone

**DOI:** 10.1155/2019/8128376

**Published:** 2019-03-28

**Authors:** Elias Estifan, Yana Cavanagh, Ashima Kapoor, Matthew Grossman

**Affiliations:** ^1^Department of Internal Medicine, St. Joseph's University Medical Center-New York Medical College, USA; ^2^Department of Gastroenterology, St. Joseph's University Medical Center-New York Medical College, USA

## Abstract

Solid pseudopapillary neoplasms (SPN) of the pancreas are extremely rare epithelial tumors with low malignant potential. They account for only 1-2% of pancreatic lesions. These masses often go unnoticed and when they become symptomatic it is often due to mass effect on neighboring structures. We encountered an unusual presentation in a healthy 34-year-old female who was found to have elevated dehydroepiandrosterone (DHEA) and testosterone levels during the evaluation of irregular menses. Subsequent abdominal imaging revealed an enhancing 2.7 cm pancreatic tail mass that was concerning for a pancreatic neoplasm. The patient underwent endoscopic ultrasound which confirmed the presence of a hypoechoic, 2.3 x 1.7 cm mass in the pancreatic tail. An intact interface was seen between the mass and adjacent structures, suggesting the absence of local invasion. Fine needle biopsy was performed and cytology was consistent with SPN. The patient later underwent curative distal pancreatectomy, with subsequent normalization of her menses. SPN are generally inactive on laboratory screening modalities (i.e., AFP, CEA, CA 19-9, and CA 125) and our patient showed no evidence of pancreatic insufficiency, pancreatic parenchymal injury, abnormal liver function, or cholestasis. Similarly to our patient, most SPN are asymptomatic. One retrospective study (spanning 15 years) reported vague abdominal pain in ~70% of patients, on initial presentation. Symptoms of tumor mass effect were the second most common. To our knowledge, this is the first reported presentation of elevated DHEA and testosterone levels associated with a solid pseudopapillary tumor in the absence of an underlying adrenal lesion or dysfunction. Despite extensive workup, no alternate etiology or correlatable medical condition could be elucidated for our patient's hormonal dysregulation. We, therefore, recommend further review and investigation into this potential correlative relationship in an effort to guide the future diagnosis and management of this unusual neoplasm.

## 1. Introduction

Solid pseudopapillary neoplasms (SPN) of the pancreas are an extremely rare epithelial tumor with low malignant potential. SPN was first described by Dr. Virginia Kneeland Frantz in 1959. It was initially known as Frantz's tumor until 1996 when the World Health Organization redefined this lesion as a “solid pseudopapillary tumor.” SPN have solid as well as papillary histological features and account for 1-2% of exocrine pancreatic tumors [1–3]. They have an overwhelming predilection for adolescent girls and young women but have rarely been reported in older females, males, and children [1, 2]. 

SPN generally present with nonspecific clinical pictures which can include abdominal or back pain, distension, and a palpable mass [3, 4]. Up to 39.2% of tumors are discovered incidentally on imaging and are asymptomatic [5]. SPN predominantly occur in the pancreas but can occur at extrapancreatic sites, such as the ovary, testis, and mesentery [6–8]. In one study on 97 patients, pancreatic SPN tumors were localized to the pancreatic head in 38.1%, pancreatic neck in 12.4%, and pancreatic body/tail in 49.5% [5]. The prognosis is generally good. Wang et al. reported that surgical resection carries low morbidity and mortality and is effective even in the presence of local invasion and metastases [5]. A recent review reported the 5-year and 10-year recurrence-free survival as 89.5% and 86.3%, respectively [9].

## 2. Case Presentation

A 34-year-old female with no notable past medical history or manifestations of hyperandrogenism underwent evaluation by her gynecologist for abnormal menstrual cycles. She described her menstrual cycle as regularly occurring every 40 to 35 days and reported it as heavy. She was found to have elevated dehydroepiandrosterone (DHEA) of 336 mcg/dL (23-266 mcg/dL), as well as borderline total testosterone of 45 ng/dL (2-45 ng/dL) and free testosterone level of 6.5 pg/mL (0.1-6.4 pg/mL), with no reported intake of exogenous androgens, hormonal birth control modalities, or glucocorticoids. Also, the patient had no known heart disease, osteoporosis, or anorexia nervosa. Endocrinology consultation and additional hormonal evaluation were unremarkable, with estradiol of 24 pg/mL (19-144 pg/mL), 17-hydroxyprogesterone of 42 ng/dL (23-102 ng/dL), FSH of 7.2 mIU/mL (2.5-10.2 mIU/mL), LH of 4.5 mIU/mL (1.9-12.5 mIU/mL), and TSH of 1.74 mIU/L (0.4-4.5 mIU/L). She subsequently underwent an abdominal computed tomography (CT) scan due to concern for an underlying adrenal adenoma. No ovarian or adrenal abnormalities were noted in imaging; however an indeterminate pancreatic tail mass measuring 2.7 x 1.3 x 2.0 cm was seen. Subsequent MRI of the abdomen further characterized the lesion as an enhancing pancreatic tail mass, concerning for pancreatic neoplasm.

**Figure 1:**
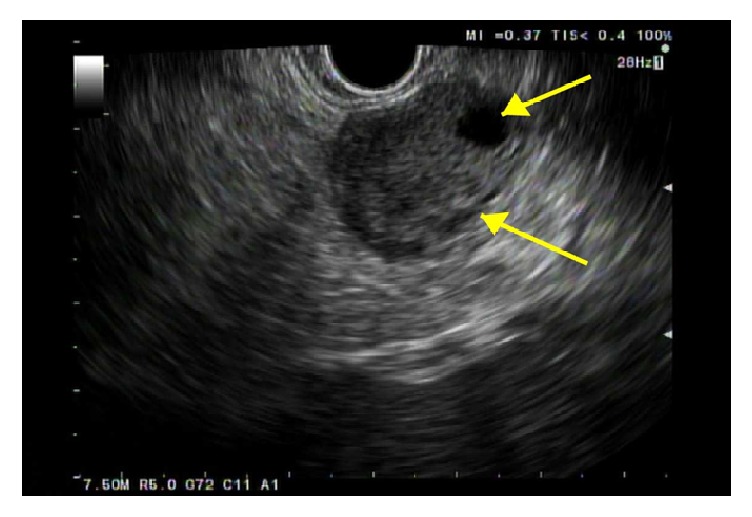
Solid mass with cystic component during an endoscopic ultrasound.

**Figure 2:**
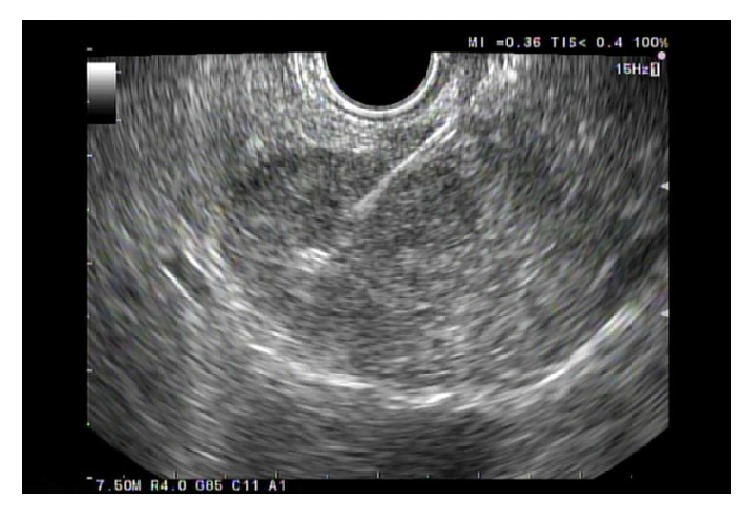
Fine needle aspiration during endoscopic ultrasound.

**Figure 3:**
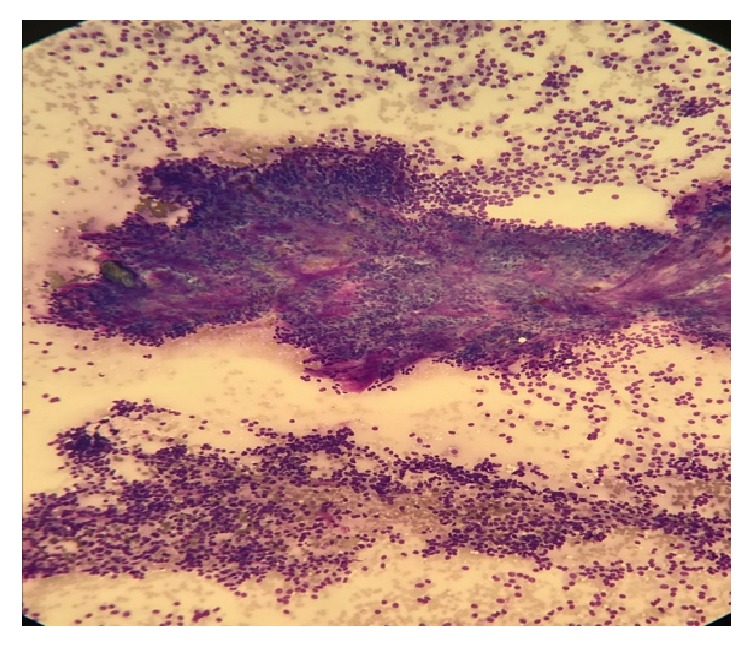
Cellular neoplasm showing papillary architecture (top) and numerous single cells also present (bottom).

**Figure 4:**
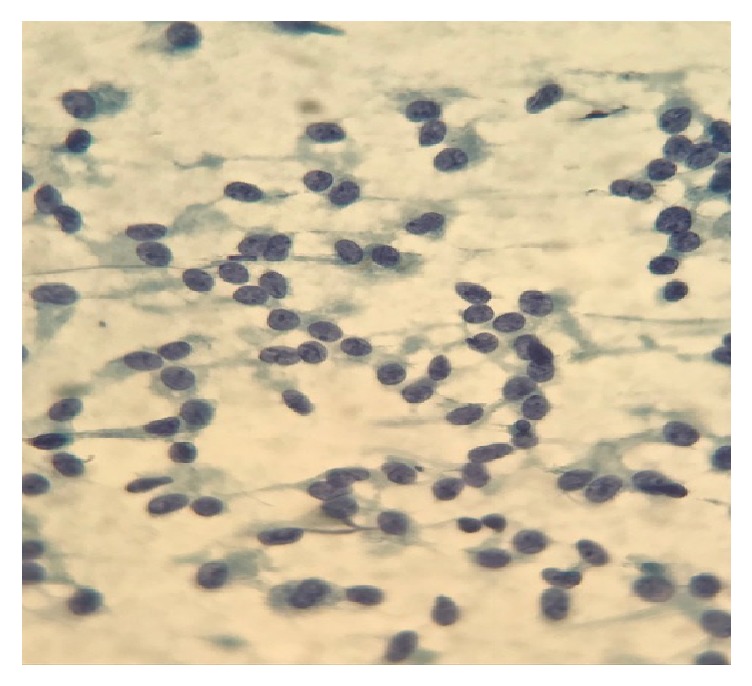
High power image shows cells with round to oval nuclei with fine chromatin and inconspicuous nucleoli and some with characteristic nuclear grooves.

**Figure 5:**
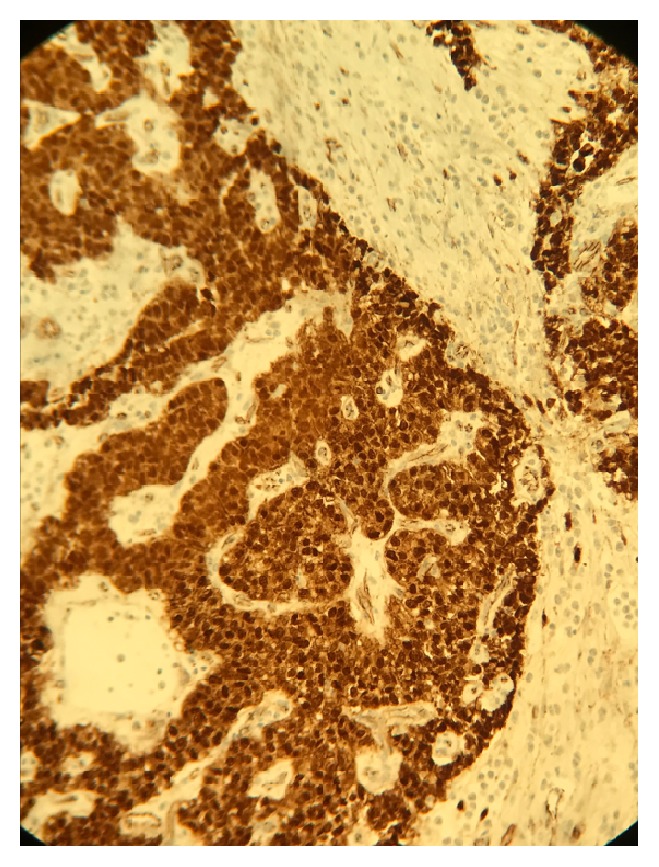
Immunohistochemical staining shows nuclear positivity for beta-catenin in the tumor cells, which is characteristic of SPN.

**Figure 6:**
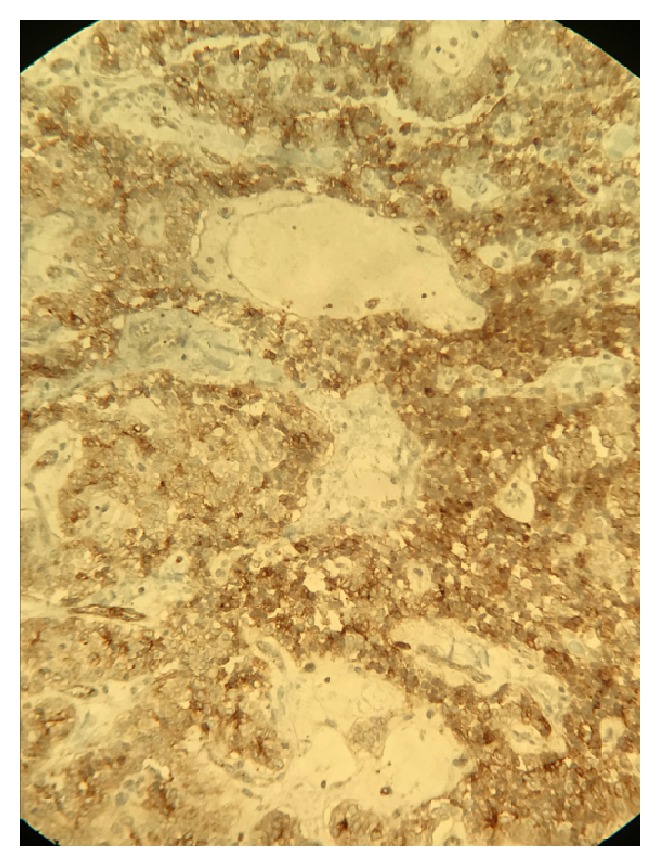
Immunohistochemical staining for CD10, showing membranous staining in the tumor cells.

**Figure 7:**
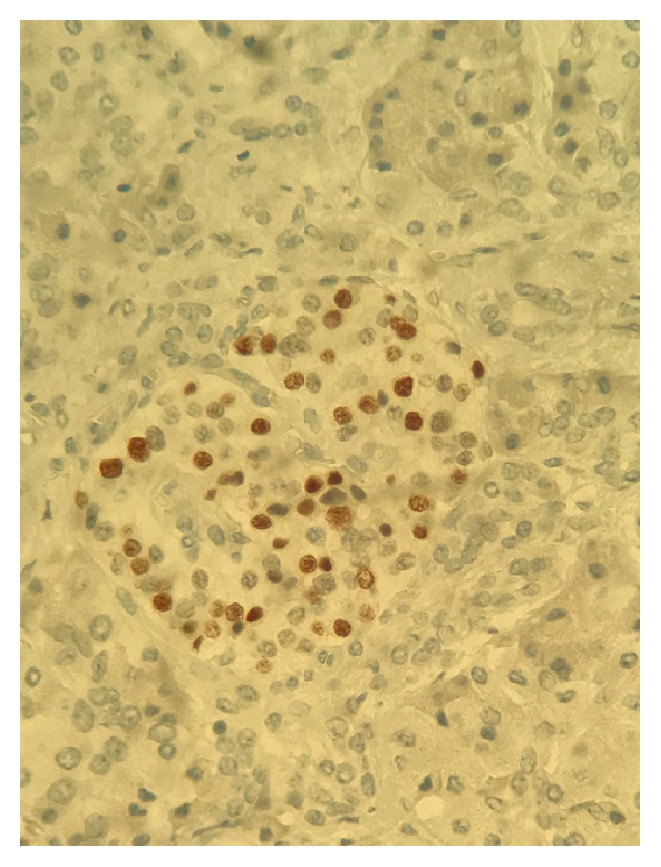
Immunohistochemical staining shows positive nuclear positivity for progesterone receptors.

The patient then underwent endoscopic ultrasound (EUS) for further evaluation and biopsy. EUS confirmed a hypoechoic mass in the pancreatic tail, measuring 2.3 x 1.7 cm (Figure 1). An intact interface was seen between the mass and adjacent structures suggesting an absence of invasion and cytology revealed SPN (Figures 2, 3, and 4). The patient was advised to undergo distal pancreatectomy and splenectomy, which she tolerated well, without any complication. Immunohistochemical staining of the resected specimen was positive for beta-catenin, CD10, and nuclear progesterone receptors (Figures 5, 6, and 7). Estrogen receptors were predominantly negative. Chromogranin and synaptophysin were negative.

Postsurgically, laboratory testing revealed normalized total testosterone of 33 ng/dL (2-45 ng/dL) and free testosterone level of 2.6 pg/mL (0.2-5.0 pg/mL); however dehydroepiandrosterone (DHEA) remained elevated at 351 mcg/dL (23-266 mcg/dL). The remaining hormonal workup was unremarkable, with estradiol of 162 pg/mL (56-214 pg/mL), 17-hydroxyprogesterone of 134 ng/dL (129-431 ng/dL), FSH of 1.7 mIU/mL (1.5-9.1 mIU/mL), LH of 3.4 mIU/mL (0.5-16.9 mIU/mL), and TSH of 1.98 mIU/L (0.4-4.5 mIU/L).

## 3. Discussion

Solid pseudopapillary tumors are often asymptomatic but may manifest vague abdominal pain or mass effect on neighboring abdominal structures. One retrospective study (conducted between 1998 and 2013) noted the most frequent presenting complaints to be vague abdominal pain in 70% of their patient base. The remaining 30% displayed signs of compression by a tumor or were asymptomatic [10]. There was no reported evidence of pancreatic insufficiency, abnormal liver function test, cholestasis, elevated pancreatic enzyme, or elevation of tumor markers in their cohort [10].

Interestingly, there is a female predilection of SPN. Multiple studies described the female predominance and female-to-male ratio of nearly 5:1 [1, 5, 9–13]. Chhabra et al. attributed the underlying female predominance to a relationship with sex hormones (i.e., estrogen and progesterone); however, their role in neoplasm growth and histogenesis remains debated [14]. Similarly to our case, progesterone receptors are identified on most SPN tumors, while estrogen receptors are rarely present [15]. Furthermore, there are no significant qualitative differences in sex hormone–receptor status between SPN obtained from male and female patients [12, 14, 15]. Interestingly, however, the rate of SPN growth is accelerated in pregnancy suggesting a hormonal basis [15].

One common cause of hyperandrogenism is polycystic ovary syndrome (PCOS) [16]. In women with PCOS ovarian androgens increase and up to 50% also have elevated DHEA levels. Another study of 611 women aged 25-50 years explored the association between testosterone concentrations and body composition and found an interesting positive dose-response relationship. As such, increased testosterone has been associated with obesity and PCOS. Although testosterone and sex hormone bending globulin (SHBG) may play an important role in the development of menstrual irregularities in obese women, our patient's BMI was only 24 [16, 17]. High blood glucose is an alternate risk factor for hypogonadism as it is associated with reduced LH states, in which testosterone levels are reduced [18]; however, this is in contrast to our patient who had elevated testosterone levels. Androstenedione is normally produced by the adrenals and to a lesser extent by the testis. Androstenedione can be interconverted to testosterone by an enzyme present in the pancreas based on a canine model. Interestingly, one study reported that androstenedione was significantly higher in patients with pancreatic cancer, while testosterone levels were significantly lower in their examined population. The increased androstenedione was associated with pancreatic cancer and may have been produced by the pancreatic tumors in their examined population [19]. However, this is, again, in contrast to our patient who had elevated testosterone.

This case appears to be the first documented presentation of SPN in concomitance with elevated DHEA and testosterone level, in an otherwise asymptomatic female. Our patient was not on hormonal birth control and endocrine evaluation revealed no alternate etiology to explain her hormonal dysregulation. The patient remains asymptomatic at one-year follow-up with mitigation of functional endogenous androgen effect and normalization of her menstrual cycle. Although there is a reported female predominance of SPN, the underlying role of sex hormones (i.e., estrogen and progesterone) in SPN growth and histogenesis remains theoretical. We recommend further review and investigation into this interesting potential correlative relationship in an effort to guide future diagnosis and management of this unusual neoplasm.
